# Chemical, molecular and structural studies of *Boswellia* species: β-Boswellic Aldehyde and 3-*epi*-11β-Dihydroxy BA as precursors in biosynthesis of boswellic acids

**DOI:** 10.1371/journal.pone.0198666

**Published:** 2018-06-18

**Authors:** Ahmed Al-Harrasi, Najeeb Ur Rehman, Abdul Latif Khan, Muhammed Al-Broumi, Issa Al-Amri, Javid Hussain, Hidayat Hussain, René Csuk

**Affiliations:** 1 Natural and Medical Sciences Research Center, University of Nizwa, Birkat Al Mauz, Nizwa, Oman; 2 Department of Biological Sciences & Chemistry, University of Nizwa, Birkat Al Mauz, Nizwa, Oman; 3 Martin-Luther-University Halle-Wittenberg, Organic Chemistry, Halle (Saale), Germany; College of Agricultural Sciences, UNITED STATES

## Abstract

The distribution and biosynthesis of boswellic acids (BAs) is scarce in current literature. Present study aims to elucidate the BAs biosynthetic and its diversity in the resins of *Boswellia sacra* and *Boswellia papyrifera*. Results revealed the isolation of new (3β, 11β-dihydroxy BA) and recently known (as new source, β-boswellic aldehyde) precursors from *B*. *sacra* resin along with α-amyrin. Following this, a detailed nomenclature of BAs was elucidated. The quantification and distribution of amyrins (3-*epi*-α-amyrin, β-amyrin and α-amyrin) and BAs in different *Boswellia* resins showed highest amyrin and BAs in *B*. *sacra* as compared with *B*. *serrata* and *B*. *papyrifera*. Distribution of BAs significantly varied in the resin of *B*. *sacra* collected from dry mountains than coastal trees. In *B*. *sacra*, high content of α-amyrin was found in the roots but it lacked β-amyrin and BAs. The leaf part showed traces of β-ABA and AKBA but was deficient in amyrins. This was further confirmed by lack of transcript accumulation of amyrin-related biosynthesis gene in leaf part. In contrast, the stem showed presence of all six BAs which are attributed to existence of resin-secretory canals. In conclusion, the boswellic acids are genus-specific chemical constituents for *Boswellia* species albeit the variation of the amounts among different *Boswellia* species and grades.

## Introduction

**Fig 1:**
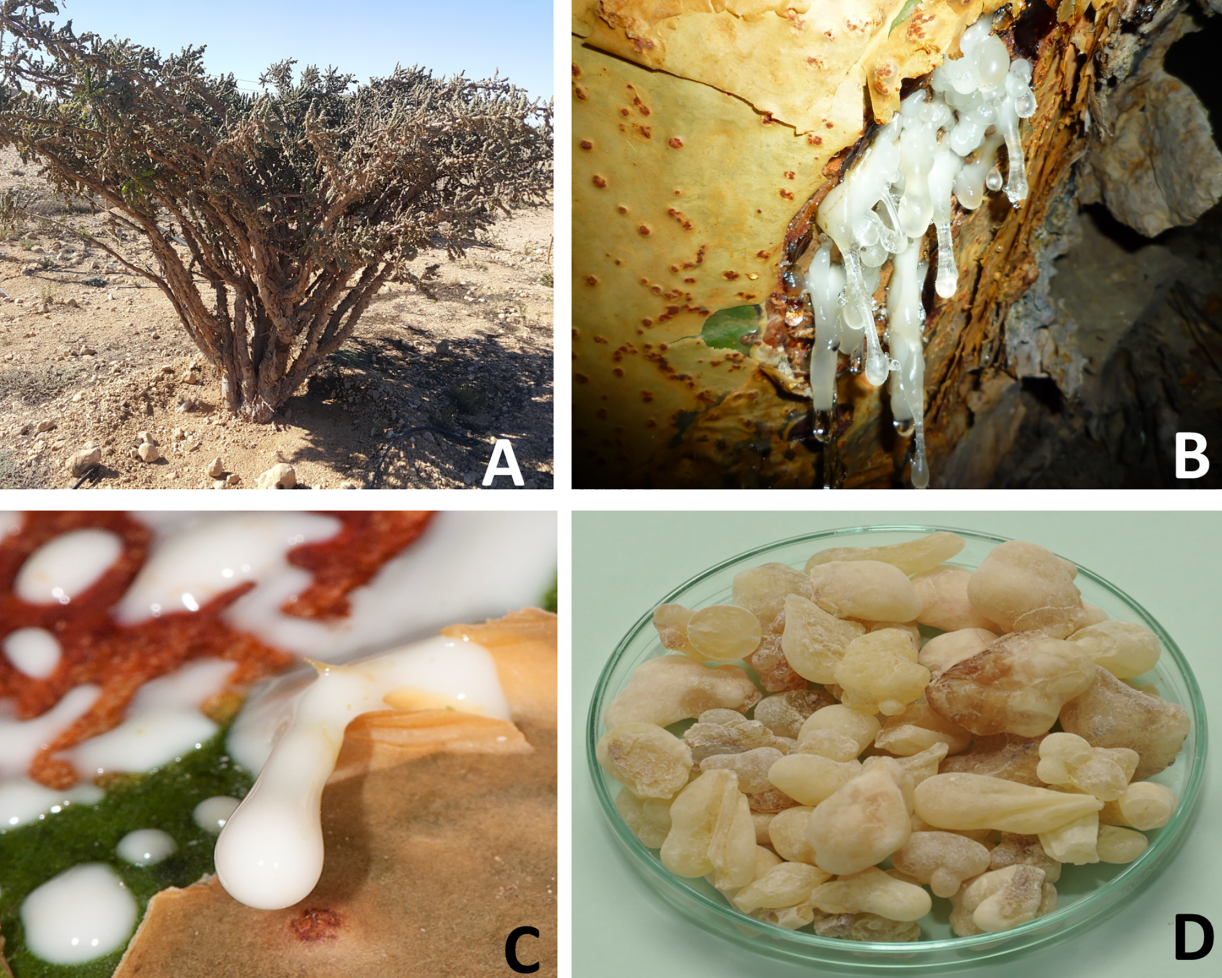
Resin extraction from *B*. *sacra* tree. a) *B*. *sacra* tree, Dhofar, Oman, b) milky substance is oozing out after the first incision, c) frankincense after a few days of incision, d) the final product of frankincense. All photos are from the authors’ lab.

Over the centuries, humanity has known and utilized frankincense since ancient times for religious, social and therapeutic purposes [1]. *Boswellia sacra* Flueck (Burseraceae) is an endemic tree to Oman and an economically important species of genus *Boswellia* [2, 3]. *B*. *sacra* is a good source of high quality frankincense and bioactive compounds having a wide-range of vital biological activities. The frankincense, a yellowish-brown oleo-gum resin and its essential oil have been well-known for their ameliorative effects against human ailments such as analgesic [4], hepato-protective [5], antioxidant [6], Alzheimer’s [7], diuretic [8], anti-coagulant [9], tumor suppressive [10], anti-inflammatory [11], cardio-protective [12], gastric, hepatic, and skin disorders [13–16]. In addition, the frankincense from *Boswellia sacra* is highly demanded for various commercial products such as cosmetic ingredients (soaps, lotions, and ointment formulation), food flavors, and perfumes [17]. Frankincense exudes from the bark of the tree after a series of man-made incisions about surface area of 10 cm^2^, at a depth of ~5 mm and 6–8 incisions in a season [18]. The frankincense oozes out from bark in the form of milky substance and gets solidified by exposure to high wind and heat (Fig 1). With the increase in demand for frankincense, the number of wounding sites can be increased, thereby affecting tree growth, physiology and regeneration capacity [19, 20]. Unsustainable wounding can further lead to pathogenic infection and insect attacks causing tree die back. The seasonal yield of frankincense per tree depends on tree size and age, as well as seasonal conditions. For example, each season an estimated 3,000 kg of resin is obtained from approximately 500,000 trees [21]. Dhofar province in Oman is the location of the highest quality of frankincense and has been involved in trading of frankincense since ancient times. It has been reported that ca. 3,000 tons were shipped to the Mediterranean countries [22, 23]. Therefore, *B*. *sacra* tree is considered one of the economic resources of Oman [24].

The frankincense resin is composed of essential oil (5–15%), mucus-like cluster (12–23%) and a lipophilic part (55–66%), however, this varies across different species and different grades [25]. The lipophilic part comprises of rich terpenoids among which are the medicinally important group of boswellic acids (BAs) [26–29]. The chemistry of BAs was commenced through a cluster of compounds isolated by Winterstein and Stein [30]. This was followed by elucidation of structure relationship between the BAs and their amyrins precursors [31, 32]. The configuration of the hydroxyl group at C-3 and the carboxylic group of BAs [33]. Using organic synthesis approaches, several studies were performed to assess the structure of BAs analogs [34, 35]. Furthermore, the chemistry and mechanism of inhibition [36–39] of BAs have helped to understand the composition and nature of this resin [40–42]. Numerous studies on the chemistry and biology of frankincense were carried out [7, 43–47]. In a recent study, we proposed robust method for quantification of BAs from *B*. *sacra* resin [44, 45]. However, the biosynthesis of triterpenes via the enzymatically driven cyclization of oxidosqualene [48] has not been fully elucidated and least has been known till now. The *B*. *sacra* tree and its physiological mechanism behind the production of resin and its essential constituents of resin have not been fully understood. In the current study, it was aimed to isolate and characterize pentacyclic triterpenes that support and further elucidate the biosynthetic pathway of BAs and to understand the variations in BAs in different parts of the *B*. *sacra* tree. In addition, molecular and anatomical analyses were used to further understand the resin production by *B*. *sacra*.

## Materials and methods

### Plant and resin collection

The ole gum resins and different parts of *B*. *sacra* were collected from the various locations in Oman (Dowkah valley- N19°07.76’ E054°25.43; Adonab—N17°20.47’ E054°04.51). The fresh plant samples were transported to lab in liquid nitrogen, whereas, the resins were brought to lab in air tight zip-bags. The resin and collected plant samples were identified by taxonomist, Saif Al-Hathmi, Royal Botanical Garden, Oman. A voucher specimen (No: BSHR-01/2012) was deposited with the Herbarium of the Chair of Oman’s Medicinal Plants and Marine Natural Products, University of Nizwa, Nizwa, Oman.

### Extraction and purification of precursors from *Boswellia sacra*

The air-dried powdered resin (1.5 kg) of *B*. *sacra* was extracted with methanol (MeOH; 3 L) at room temperature (27 **°**C; 3 × 15 days) and evaporated under reduced pressure to yield a crude MeOH extract (1.3 kg). The crude methanol extract was subjected to silica gel column chromatography (SiO_2_; 500 g; 70–230 mesh; Merck) with gradient increasing polarity of *n*-hexane/ethyl acetate (EtOAc) and finally washed with pure EtOAc to afford twelve fractions (BS_1_-BS_12_). Fraction four (BS_4_), 20% *n*-hexane/EtOAc, was further loaded on a column chromatography using gradient mobile phase of 10, 20, 30 and 50% EtOAc/*n-*hexane to get four sub fractions (BSS_1_-BSS_5_). After taking TLC, sub-fractions two (BSS_2_) and three (BSS_3_) were combined, eluted with 20 and 30% EtOAc*/n*-hexane, and loaded over column chromatography using isocratic mobile phase of 30% EtOAc/*n*-hexane to afford compound **10** and 40% EtOAc/*n*-hexane attributed compound **19**. Both compounds were found to be new after studying detail spectroscopic techniques.

### Characterization of purified compounds

#### Compound 1

Colorless solid; ${\left[\alpha \right]}_{D}^{25}=+83.3$ (MeOH, c = 0.024); **mp:** 184‒186˚C; **IR** (solid) **υ**_***max***_ = 3440 (OH), 2940, 2870, 1730 (CHO), 1450, 1370, 1225, 1160, 1025, 970, and 880 cm^1^; **HR ESI-MS** = 441.3722 [M + H;C_30_H_48_O_2_]; ^**1**^**HNMR** (CDCl_3,_ 600 MHz) = δ 9.71 (1H, s, H-24), 5.12 (1H, t = 3.0 Hz, H-11), 4.11 (1H, br. s, H-3), 1.10 (3H, s, H-23), 1.08 (3H, s, H-28), 1.02 (3H, s, H-26), 0.90 (3H, d = 6.0 Hz, H-30), 0.82 (3H, s, H-25), 0.78 (3H, s, H-28), 0.77 (3H, d = 6.0 Hz, H-29); ^**13**^**C-NMR** (CDCl_3_, 125 MHz): δ 204.7 (C-24), 139.6 (C-13), 124.3 (C-12), 69.2 (C-3), 59.1 (C-18), 52.2 (C-4), 49.2 (C-5), 46.3 (C-9), 42.3 (C-8), 41.5 (C-22), 40.0 (C-14), 39.6 (C-19), 39.5 (C-20), 37.2 (C-10), 33.7 (C-17), 33.2 (C-1), 33.1 (C-7), 31.2 (C-21), 28.7 (C-28), 28.0 (C-15), 26.4 (C-16), 25.8 (C-2), 23.5 (C-11), 23.2 (C-27), 21.3 (C-30), 19.7 (C-23), 17.8 (C-6), 17.4 (C-29), 17.0 (C-26), 14.2 (C-25).

#### Compound 5

White solid; **UV(MeOH)λ**_***max***_ = 220 (3.87); **mp:** 187‒189˚C; **IR (solid) υ**_***max***_ **cm**^**-1**^: 3420, 2965, 2864, 1702, 1668, 1632, 1467, 1319, 1264, 1228, 970 and 880; ${\left[\alpha \right]}_{D}^{25}=+34{}^{\circ}$ (MeOH, c = 0.40); **ESI-MS (*rel*. *int*.):***m/z* 495.93 [M + H]^+^ (C_30_H_48_O_4_); 476.97 [(M—H_2_O + Na)+]; ^**1**^**H NMR** (CDCl_3,_ 600 MHz): δ 5.35 (1H, *d*, 3.0 Hz, H-12), 4.53 (1H, *dd*, 10.2, 3.0 Hz, H-11), 3.24 (1H, dd, 11.4, 4.8 Hz, H-3), 2.31 (1H, m, H-1), 2.07 (1H, *dd*, 16.0, 2.0 Hz, H-16), 2.01 (1H, m, H-2), 1.88 (1H, m, H-15), 1.81 (1H, d, 10.2, H-9), 1.68 (1H, *dd*, 14.1, 3.0, H-6), 1.55 ((1H, m, H-2),1.54 (1H, *dd*, 11.8, 3.1 Hz, H-5), 1.46 (2H, m, H-7/22), 1.42 (1H, m, H-21), 1.39 (2H, *d*, 8.9 Hz, H-18/1), 1.37 (1H, m, H-21), 1.32 (1H, m, H-7), 1.30 (1H, m, H-6), 1.28 (2H, m, H-22), 1.24 (3H, s, H-23), 1.21 (1H, m, H-19), 1.18 (1H, m, H-15), 1.14 (3H, s, H-26), 1.13 (1H, *dd*, 16.0, 2.0 Hz, H-16),1.05 (3H, s, H-27), 1.03 (3H, s, H-25), 0.98 (3H, *d*, 6.6 Hz, H-30), 0.91 (1H, m, H-20), 0.88 (3H, *d*, 6.5 Hz, H-29), 0.78 (3H, s, H-28); ^13^C-NMR (CDCl_3_, 125 MHz): δ 177.5 (C-24), 146.3 (C-13), 124.5 (C-12), 81.8 (C-11), 78.8 (C-3), 58.4 (C-18), 55.7 (C-5), 49.0 (C-9), 43.1 (C-4), 42.0 (C-14), 41.3 (C-22), 40.0 (C-8), 39.4 (C-19), 39.3 (C-20), 37.8 (C-10), 35.5 (C-1), 33.6 (C-17), 33.4 (C-7), 31.1 (C-21), 28.1 (C-28), 27.8 (C-15), 27.2 (C-2), 26.7 (C-16), 22.0 (C-23), 21.3 (C-30), 18.2 (C-6), 18.1 (C-27), 16.6 (C-26), 15.6 (C-29), 13.5 (C-25).

### HPLC-DAD analysis

The BAs amount was quantified by a parallel liquid chromatography (1260 HPLC, Agilent Technologies; Japan) connected with a reverse phase C18 column (3.9 × 150 mm; Waters; Nova-Pak, USA). The samples were dissolved (10 mg·mL^-1^) in HPLC-grade methanol, and then properly diluted. The chromatographic separation was carried out at a constant flow rate of 1 mL min^-1^ with the following conditions: Mobile phase A = 99.9% water with 0.1% acetic acid; Mobile phase B = 99.9% acetonitrile with 0.1% acetic acid; Total running time (stop time) = 30 mints for BAs and 35 mints for amyrins; Washing time (post time) = 1 minute; Injection volume = 20 μL; Column type = C18, Waters; Column temperature = 40 ⁰C; DAD Signal = wave length of 254 for BAs and 210 for amyrins; Band width = 4.0; Reference wave length = 254 nm, Reference bandwidth = 100 nm.

### RNA extraction and RT-PCR

RNA was extracted from the leaf parts of the *B*. *sacra* tree according to the protocol of Chen et al. [49] with some modifications. Briefly, the leaf (1.0 g) was ground to powder in chilled mortar and pestle in liquid nitrogen and was immediately transferred to RNase and DNase free falcon tubes having extraction buffer (NaCl-0.25 M, Tris-HCl—0.05M, pH 7.5; EDTA -20 mM, sodium dodecyl sulphate-1% and PVP-4% as described by Chan et al. [49]. RNA with good integrity and purity was used to synthesize cDNA through DiaStar™ RT kit (SolGent, Korea) according to the provided manufacturer’s standard protocol. The gene expression of α-amyrin synthase (Forward 5’-CTTTGTGGTCCTGCTGGTAA-3’; Reverse 3’-TGGCTTCACATTTGGAAGAG-5’) and *Squalene-synthase* (Forward 5’-GAGGCACCCAGATGATCTTT-3’; Reverse 3’-GAGCGAAACTTCTGGAGACC-5’) was further evaluated through RT-PCR. A 2x Real-time PCR Kit (BioFACT^TM^ Korea) including 10nM of each gene specific primer with 100 ng of template cDNA in a final volume of 20 μL reaction mixture was used. The whole reaction was carried out according to manufacturer’s standard protocol using Eco™ Real-Time PCR (Illumina™ USA) with a “no template control” (NTC) as a negative control. The expression of each gene was compared with relative expression of Actin as internal control and the experiment was repeated three times.

### Microscopic analysis of stem

The bark samples collected from *B*. *sacra* tree (epidermal and cambium region; 20 mm^2^) were fixed in Karnovsky solution (Karnovesky = 2.5% gluteraldehyde in sodium. cacodylate buffer and Gluteraldehyde) dehydrated in increasing ethanol series [30, 50, 70, 90, and 100% (three times)] and then infiltrated with resin ethanol for polymerization [acrylic resin glycolmethacrylate and 100% ethanol at a ratio of 1:1 and then pure resin]. The blocks were cut on a microtome into 10um size. Similarly, samples for SEM were fixed, dried to the critical point with CO_2_ and gold sputtered prior to observation. Details about light microscopy and scanning electron microscopy (SEM) are described by Peckys et al. [50].

### Statistical analysis

The quantification of BAs and RT-PCR related analysis were performed in triplicate and represented with standard deviation. The Non-metric multidimensional scaling (NMDS) which analyses the quantities of BAs in different parts of the tree and different kinds of resin, was performed by PAST (v3.01; University of Auckland, New Zeeland). One-Way ANOVA analysis was performed using Graph Pad prism (v6.01) to identify the significant samples.

## Results and discussion

### Isolation and characterization of β, 11β-dihydroxy boswellic acid

**Fig 2:**
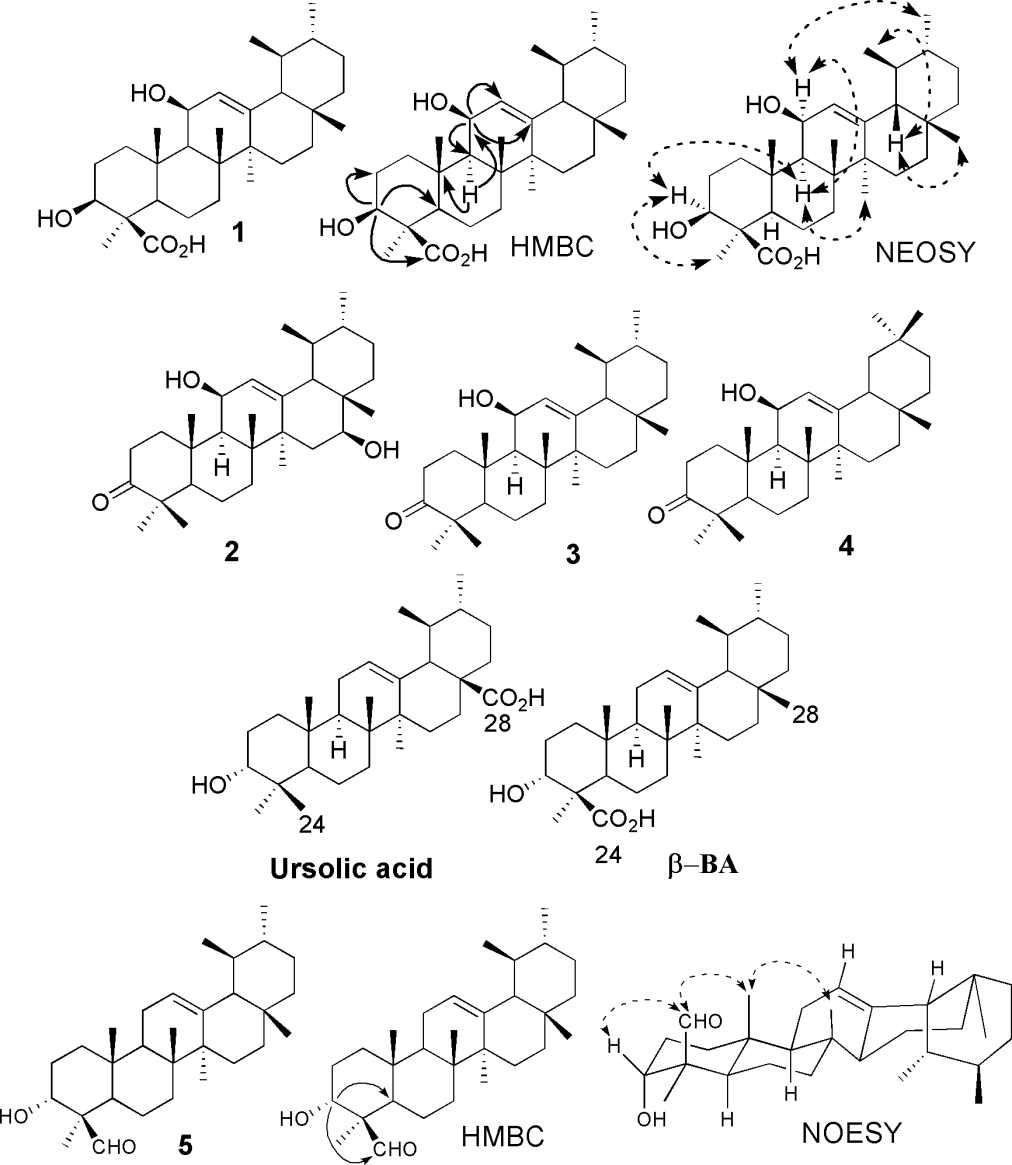
Chemical compounds and their NMR configuration compound 19 and its HMBC, COSY and NOESY correlations; Compounds 20–22 isolated from *Protium kleinii* [54]; Ursolic acid and β-BA; Compound 10 and its HMBC and NEOSY correlations.

We have isolated 3β, 11β-dihydroxy boswellic acid **1** (Fig 2) for the first time from the resin of *B*. *papyrifera*. It analogue (**15)** was previously isolated for the first time from *B*. *sacra* [51]. Compound **1** was isolated as white amorphous powder having molecular formula of C_30_H_48_O_4_ and evidenced by ESI-MS which exhibited molecular ion peaks at 495 [M + Na]^+^ and 477 [M^+^–H_2_O + Na] (7˚ of unsaturation). The IR spectrum of **1** showed characteristic absorptions bands for hydroxyl (3410 cm^-1^), carboxylic acid (1702 cm^-1^), and a double bond (1632 cm^-1^) functional groups. The ^1^H NMR spectrum of **1** displayed five tertiary methyls (δ_H_ 1.24, 1.14, 1.05, 1.03, 1.06 and 0.78, each single), two secondary methyls (δ 0.98, d, *J* = 6.6 Hz and 0.88, d, *J* = 6.5 Hz) and a trisubstituted olefinic proton (δ 5.35, d, *J* = 3.0 Hz), which are characteristic of ursane-type triterpenes related to boswellic acids [52, 53]. The ^13^C NMR spectrum (experimental part) of compound **1** displayed 30 distinct peaks accounted for by seven methyls, eight methines, eight methylenes and seven quaternary carbons. The ^13^C-NMR spectrum of **1** (experimental part) also showed the presence of an olefinic group at δc 124.5 and 146.3 (C-12, C-13), a carboxylic group at δc 177.5 (C-24), and two hydroxylated carbons at δc 78.8 and 81.8 (C-3, C-11). All the positions of the substitutions were deduced using the COSY and HMBC 2D NMR techniques (Fig 2). Compound **1** is direct precursor of the 3-*epi*-β-Boswellic acid and its known diastereomeric analogue 3α, 11α-dihydroxy boswellic acid **15**.

The ^1^H NMR spectrum confirmed the presence of hydroxyl group at C-3 and was in β-orientation as evidenced by the doublet of doublet (11.4, 4.8 Hz) of the α-positioned geminal proton which appeared at δ_H_ 3.24, an interpretation and β-orientation further substantiated by HMBC correlation between H-5 (δ_H_ 1.54) and C-3 (δc 78.8) and NEOSY correlation between H-3 and CH_3_-23 position. On the other hand, the singlet peak at δ 5.52 (H-12) correlated with C-11(δc 81.8), C-9 (δc 49.0), C-13 (δc146.3) and C-14 (δc 42.0) in the HMBC spectrum allowed its assignment to olefinic double bond between C-12 and C-13.

Besides olefinic double bond, the ^1^H NMR spectrum of **1** displayed a peak at δ_H_ 4.53 previously observed in boswellic acid derivatives [51]. The three bond long range correlations from this signal to C-9 (δc 49.0), C-12 (δc 124.5), and C-13 (δc 146.3) in the HMBC spectrum allowed its assignment to H-11. This proton was associated with a carbon signal at *δ* 81.8 in the HSQC spectrum and showed COSY correlation with a signal at δ_H_ 1.81 (H-9) and 5.52 indicating the location of hydroxyl group at C-11. The configuration of H-11 in **1** was determined to be axial on the basis of coupling constants with H-9 and H-12 and further supported by NOESY correlation between H-11 and CH_3_-25which is in close agreement with the reported compounds **2**–**4** (Fig 2) [51]. One known analogue **15** has also been isolated from the same species having^13^C-NMR at δ 70.6, and ^1^H-NMR at δ 4.24 (dd, *J* = 9.6, 3.0 Hz) corresponded to the C-11 position. However, the downfield shift observed in the ^13^C-NMR of compound **1** at δc 81.8 further strengthens the β-orientation of hydroxyl group at C-11 position [54].

Similarly, the H-3 showed an HMBC cross-peak with a peak at δc 177.7 which further supported the presence of carboxylic group in ring A at either C-23 or C-24 positions. The characteristic NOESY correlation between CH_3_-23 and H-5 allowed the assignment of the functional group at C-24 as previously observed boswellic acids [55]. All the above data can be accommodated only on an urs-l2-ene triterpenoid structure for compound **1** with a hydroxyl group at the C-3β position [56] and another secondary hydroxyl group attached to the C-l lβ position [54]. Thus, on the basis of spectroscopic data and also comparison with literature data, structure of compound **1** was deduced as 3β, 11β-dihydroxy-12-en-24-oic acid commonly known as 3β, 11β-dihydroxy-β-boswellic acid.

It is noteworthy mentioning that the sole source of boswellic acids is *Boswellia* species where the oxidation takes place at C-24 by Cytochrome P450 enzymes [57] whilst ursolic acid exists abundantly in plant kingdom and the oxidation takes place at C-28. Thus, the boswellic acids can be considered as genus-specific and can be specific biomarkers for *Boswellia* species albeit the variation of the amounts among different *Boswellia* species and grades (Fig 2).

### Isolation and characterization of boswellic aldehyde

Compound **5** was obtained as a colorless solid having melting point of 184‒186˚C and assigned a molecular formula of C_30_H_48_O_2_ as evidenced by HRESI-MS which showed an [M + H]+ ion peak at *m*/*z* 441.3722 (calculated: 441.3718). The ESI-MS molecular ion peak suggested seven double bond equivalents, six of which are assigned to the rings of the pentacyclic triterpene skeleton and one was attributable to the formyl group (δ_C_ 204.6). The IR spectrum of **5** showed characteristic peaks at 3440, 2940, 2870 and 1730 cm^-1^, which confirmed the presence of OH and CHO groups. An equivalent, assigned to a double bond consistent with Δ^12^-ursane skeleton, showed resonances at δc 124.3 (C-12) and 139.6 (C-13).

In the ^1^H NMR spectrum (experimental) of compound **5**, the presence of vinylic proton at 5.12 (1H, t, *J* = 3.0 Hz, H-12), one oxymithine proton at 4.11 (1H, br s, H-3), five tertiary methyls at 1.10, 1.08, 1.02, 0.82, and 0.78 (all singlets), and two secondary methyls at 0.90, (3H, d, *J* = 6.0 Hz) and 0.77 (3H, d, *J* = 6.0 Hz) were observed, unambiguously confirming the presence of Δ^12^-ursane skeleton [51]. The ^13^C NMR spectrum (experimental part) exhibited a total of 30 carbon signals which were sorted out into nine CH_2_, seven CH_3_, seven CH including one olefinic, and one oxygen-bearing carbon as well as seven quaternary carbons. Comparison of the ^1^H and ^13^C NMR spectra of compound **5** with those of β-boswellic acids [55] displayed close similarities with notable difference being the substitution mode in ring A. The chemical shift at δc 69.2 in the ^13^C-NMR spectra and HMBC correlations of H-3 with CH_3_-23 (δc 19.7) and formylcarbon C-24 (δc 204.7) confirmed the presence of hydroxyl group in ring A at C-3 position, while the alpha orientation was further confirmed by NEOSY cross peaks (Fig 2) [58].

**Fig 3:**
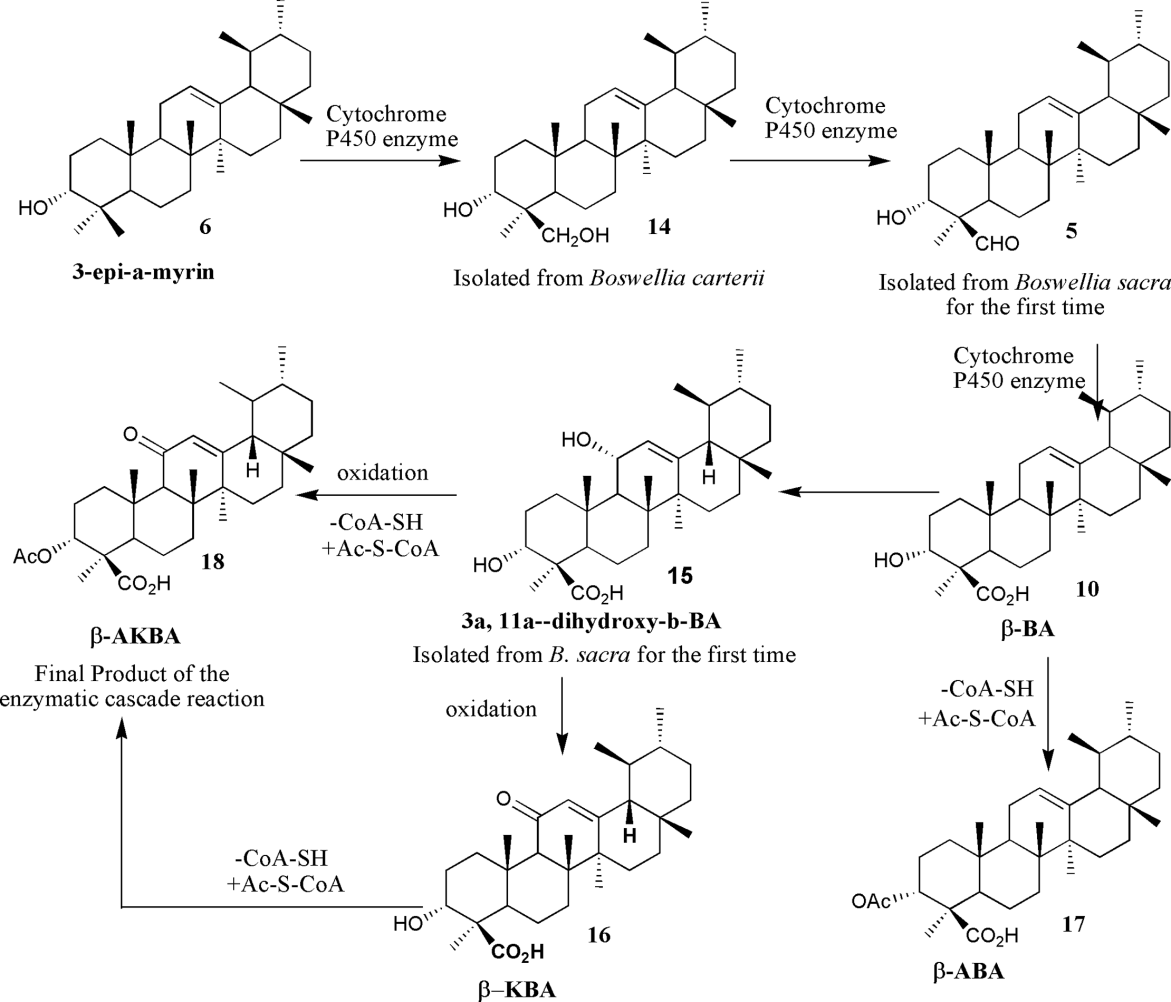
Formation of boswellic acid derivatives.

The presence of formyl group was assumed to be located either at the C-23, C-24 or at the C-28 position. The key HMBC correlations of the formyl proton H-24 (δ_H_ 9.71) with C-3 (69.2) and C-4 (52.2); CH_3_-23 (19.7) with C-3 (69.2) and C-24 (204.7) proved that aldehyde group located either at C-23 or C-24 positions. Since the formyl group was assumed to be formed in the reaction by oxidation of the CH_2_OH group of **14 (**[59]; Fig 3), it might be expected that the -CHO group will have the same location and orientation as the CH_2_OH group has in **9.** Therefore, the formyl group in compound **5** located the position at C-24 **(**β-orientation) which was further confirmed by NEOSY correlations [60]. On the basis of the above results, compound **5** was deduced as3α-hydroxy-urs-12-ene-24-al commonly named as boswellic acid aldehyde. This compound, up to the best knowledge, is described for the first time from *B*. *sacra* and recently isolated from *B*. *serrata* as a first natural product [61].

### Biosynthetic pathway of 3-*epi*-α-amyrin and its precursors

**Fig 4:**
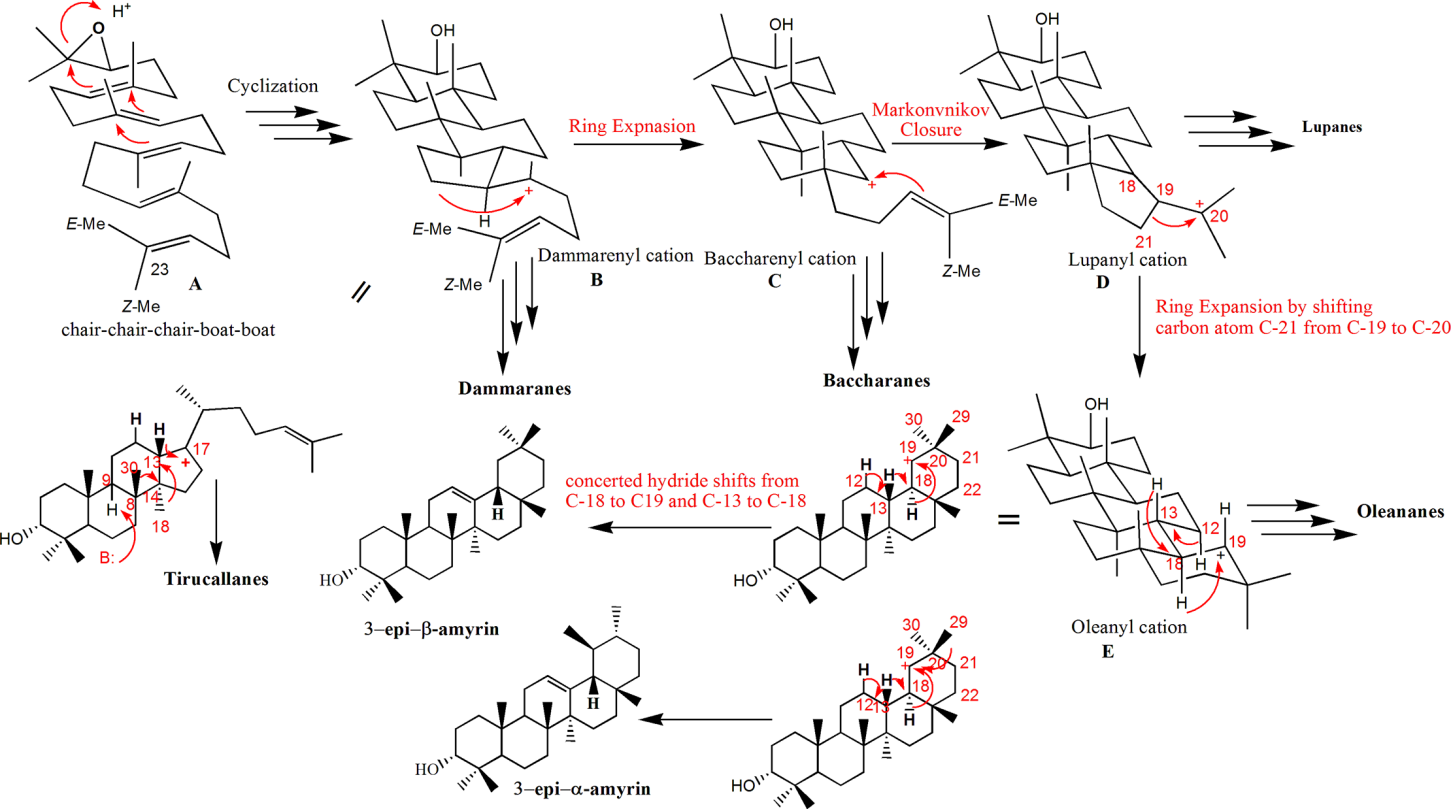
Proposed biosynthetic pathway of 3-*epi*-α-amyrin and its precursors.

The biosynthesis of β-amyrin has been postulated [62] following the first proposal of the isoprene rule [63, 64]. In order to understand the biosynthesis of BA and its consecutive oxidative and acetylated derivatives, the biosynthesis of their precursor β-amyrin is depicted in Fig 4. This historic precursor is commenced at the *chair*–*chair*–*chair*–*boat* conformation of (3*S*)-2,3-oxidosqualene **A**, which cyclized to the corresponding 6.6.6.5-fused tetracyclic dammarenyl C-20 cation **B** which at this stage led to the formation of the dammaranes isolated from *Boswellia* species [62]. A concerted hydride shifts from C-13 to C-17, methyl group (C-18) shift from C-14 to C-13 and methyl group (C-30) shift from C-8 to C-14, followed by enzymatically catalyzed base abstraction of C-9 proton, generating the double bond between C-8 and C-9, afforded tirucallanes isolated from *Boswellia* species [65]. After ring D enlargement, electrophilic addition on the terminal double bond of the tetracyclic baccharenyl cation **C**, a five-membered ring E is constructed and the Lupanyl cation **D** is generated which leads to the formation of Lupanes isolated from *Boswellia* species [66]. This is followed by ring E enlargement which occurs via C-30 shift from C-20 to C-19 to afford the oleanyl cation **E** which can lead to the formation of Oleananes isolated from *Boswellia* species [67] to which β-amyrin (precursor for α-Boswellic acid) belongs. The oleanyl cation **F** then undergoes sequential 1,2-hydride shifts from C-18 to C-19 and from C-13 to C-18 followed by elimination of H-12 to afford the 6.6.6.6.6-fused pentacyclic Ursanes isolated from *Boswellia* species [68] to which α-amyrin (precursor for β- Boswellic acid) belongs.

### Chemical diversity of boswellic acids

The four amyrins are known from different plant species including *Boswellia* [69], some of their corresponding boswellic acids remained unknown due to their existence in infinitesimal amounts in the resins which doesn’t allow their isolation and hence characterization. It is imperative to highlight a few conventions adopted in the literature which can be confusing. In general, α-amyrins where C-29 and C-30 are in vicinal relationship at C-19 and C-20 respectively will lead to β-boswellic acids. Moreover, β-amyrins where C-29 and C-30 are in germinal relationship both at C-20 will lead to α-boswellic acids.

**Fig 5:**
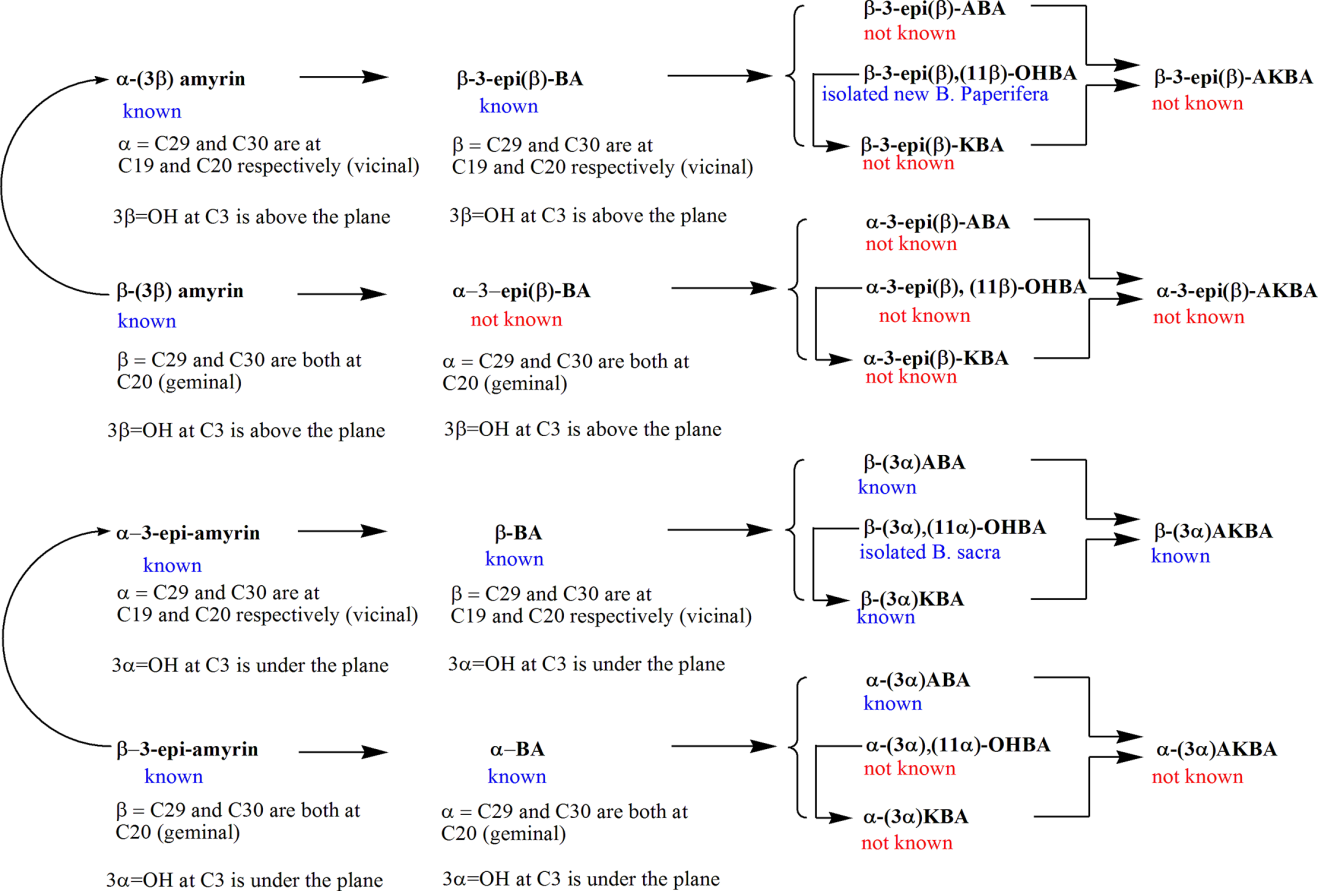
Conversions of α-amyrin and β-amyrin into boswellic acids.

C-H activation and oxidation of the α-configured methyl group C-24 of the 3-*epi*-α-amyrin **6,** the 3-*epi*-β-amyrin **8** (Fig 5) afforded the β-boswellic acid **5** and α-boswellic acid **7** respectively which were isolated from different *Boswellia* species. [70, 71] Likewise C-H activation and oxidation of the α-configured methyl group C-24 of the α-amyrin **2** furnished the 3-*epi*- β-boswellic acid **6** (where the hydroxyl group at C-3 and the methyl group at C-4 are both β-configured and the two methyl groups at C-19 and C-20 are in vicinal relationship) which was only reported once from *B*. *carteri* [59].

**Fig 6:**
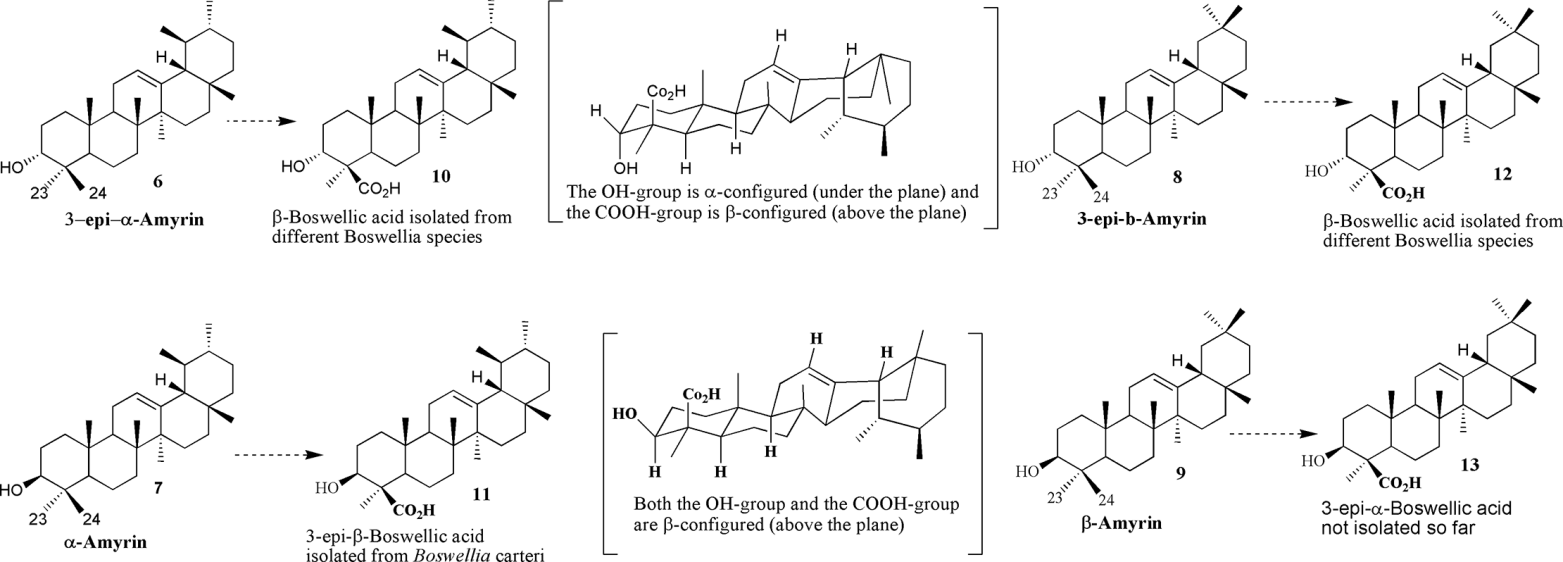
Configuration of amyrins and boswellic acids.

Similarly, C-H activation and oxidation of the α-configured methyl group C-24 of the β-amyrin **9** should afford 3-*epi*-α-boswellic acid **8**. However, as far as we are aware there is no report which describes the isolation of the 3-*epi*-α-boswellic acid. We found it vitally important to introduce the prefix “*epi*” to differentiate the configuration of the hydroxyl group at C-3 which has been a long-standing confusion in literature for decades. In this regard, 3β-amyrin where the β-OH at C-3 is above the plane will lead to 3-*epi*-boswellic acid again where the β-OH at C3 is above the plane. In contrast, 3-*epi*-amyrin where the α-OH at C-3 is under the plane will lead to boswellic acid where the α-OH at C-3 is under the plane (Fig 6).

### Biosynthesis of boswellic acids

A series of enzymatic oxidations take place to convert C-24 into carboxylic acid at 3-*epi*-α-amyrin **6** which undergoes a conversion to the corresponding alcohol **14** that is isolated from *B*. *carteri* [59]. Alcohol **14** is oxidized to the aldehyde **5** that is isolated as a new compound from *Boswellia sacra* with full spectrometric data [61]. The aldehydic group in **5** upon oxidation by Cytochrome P450 enzyme affords the carboxylic acid moiety of β-boswellic acid **10**. The OH group is introduced at C-11 of the β-boswellic acid **10** which led to the formation of diol **15** which has also been isolated from *Boswellia sacra*, as a new source, by our group and its ethoxy analogue was reported earlier from *B*. *neglecta* [51] which upon oxidation of the OH at C11 should lead to ß-KBA **16**. Acetylation of the OH at C-3 in β-boswellic acid **5** via Ac-CoA and an acetyl-transferase will give β-ABA **17**. Oxidation of the OH at C-11 and acetylation of the OH at C-3 of diol **15** afforded the AKBA derivative **18** which appeared to be the final product of this enzymatically-driven cascade reaction. The reaction sequence of the acetylation and oxidation which led to formation of ABA and KBA is not known hitherto and lacks supported evidences. Certainly, comprehensive physiological investigations on the frankincense trees are mandatory to elucidate the exact order of steps for the biosynthesis of AKBA.

**Fig 7:**
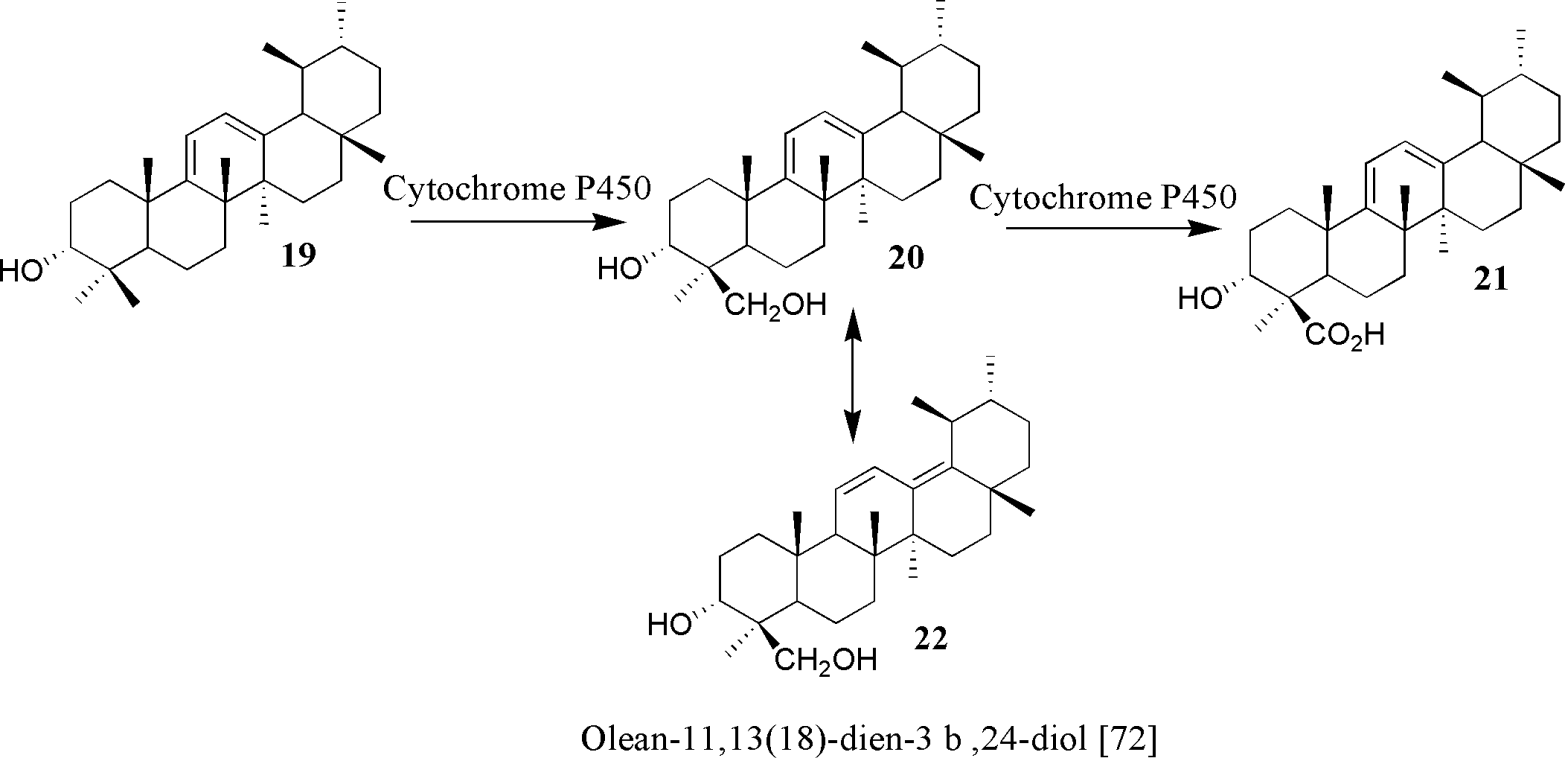
Formation of pentacyclic isomers.

The boswellic acid derivative **21** (Fig 7) have resulted from the oxidation of alcohol **20**. Isomer **22** was isolated from *B*. *sacra* earlier [72]. In a similar analogy to alcohol **14**, alcohol **20** is a result of the oxidation of the amyrin **19**. All these four derivatives are isolated by our group from *B*. *sacra* [72].

### Quantification of α-amyrin and BAs in different *Boswellia* tree parts and resins

Given the fact that there are no specific enzymes isolated from *Boswellia* species to elucidate the biosynthetic pathway, we have attempted quantification of boswellic acid derivatives using HPLC. This quantification is performed on the leaf, bark, roots and resin of *B*. *sacra* tree. Moreover, three resins are analyzed for their amyrins and BA contents namely *B*. *sacra*, *B*. *serrata* and *B*. *papyrifera*. In order to verify the influence of the geographical location of the *Boswellia sacra* tree, the resins of two grades (mountainous and costal) were analyzed for their boswellic acids composition. We realized that the quantities of the triterpenes vary significantly depending on the *Boswellia* species as well as on the environmental factors within the same species (Table 1). While in some species large amounts of a triterpenes exist, only infinitesimal quantities of the same compound can be detected which explains the huge chemical diversity of the triterpenes in *Boswellia* species (Table 1).

**Table 1:** Quantification of amyrins and BAs in different *Boswellia* tree parts.

Part	*Epi*-α-amyrin	α-amyrin	β-amyrin	Total Amyrins	β-BA	α-BA	β-ABA	β-KBA	β-AKBA	α-ABA	Total BAs
**Bark** **(Hypodermis)** **outer**	ND	ND	ND	ND	ND	ND	0.02± 0.00	Trace	0.32±0.01	0.66±0.02	1.0±0.01
**Bark** **(Cambium)** **inner**	ND	ND	ND	ND	1.34 ± 0.08	Trace	0.98±0.02*	0.14±0.01	1.37±0.04*	1.10±0.1	4.93±0.37**
**Root**	-	2.63 ± 0.02	-	2.63 ± 0.03	ND	ND	ND	ND	ND	ND	ND
**Leaf**	-	ND	-	ND	ND	ND	0.28 ± 0.01	ND	0.28±0.01	ND	0.56 ± 0.01

The values with± shows the standard error of mean values of three replications. The asterisks (

*p<0.05

**p<0.01) show that the values are significantly different in each column using a One-Way ANOVA analysis in Graph Pad prism (v6.01) using Bonferroni test.

*B*. *serrata* is distinguished from the other two species by higher amounts of β-KBA (1.29%) whereas *B*. *papyrifera* and *B*. *sacra* possess 0.46% and 0.73% respectively. This suggests β-KBA as a biomarker for *B*. *serrata*. *B*. *papyrifera* proved a complete absence of β-BA and lower amounts in case of *B*. *serrata* whilst higher amounts on average of the two grades in *B*. *sacra*. Therefore, β-BA can be identified as a biomarker for *B*. *sacra*. Comparable quantities of α-BA in all three species are found which makes it difficult to use α-BA as a biomarker to distinguish these species. The same conclusion can be drawn for α-ABA and β-ABA. The amounts of β-AKBA are comparable in *B*. *serrata* and *B*. *papyrifera* as well as in the costal grade of the *B*. *sacra* whereas higher amounts are observed in the mountainous type of *B*. *sacra*. The precursor of all β-boswellic acids (β-BA, β-ABA, β-KBA and β-AKBA) presented in Table 1 above is *epi*-α-amyrin which was detected in higher amounts in *B*. *sacra* species whilst completely absent in *B*. *serrata* and *B*. *papyrifera*. The total β-Boswellic acids content is 7.04 and 9.68% in *B*. *papyrifera* and *B*. *serrata* respectively (Table 2). Significantly higher amounts of β-boswellic acids are observed for the two *B*. *sacra* species. It is noteworthy mentioning that the amounts of α-ABA are lower in the case of *B*. *sacra* when compared with the other two species.

**Table 2:** Quantification of α-amyrin and BAs in different kinds of *Boswellia* resins.

Resin source	*Epi*-α-amyrin	α-amyrin	β-amyrin	Total Amyrin	β-BA	α-BA	β-ABA	β-KBA	β-AKBA	α-ABA	Total BAs
*B*. *papyrifera*	Trace	Trace	Trace	Trace	ND	2.36±0.03	0.57±0.04	0.46±0.02	3.65±0.11	1.84±0.01	8.88±0.23
*B*. *serrata*	trace	1.49±0.01	0.98±0.01	2.47±0.13	0.61±0.02	2.31±0.11	0.97±0.1	1.29±0.13*	4.42±0.21	1.92±0.16	11.52±0.27
*B*. *sacra* Shaabi (costal)	5.80±0.20**	1.38±0.03	1.24±0.12*	8.42±0.23	1.73±0.21	4.34±0.28**	0.12±0.01	0.73±0.01	3.82±0.11	0.60±0.01	11.34±0.62
*B*. *sacra* Green (mountainous)	2.67±0.21	12.10±0.7***	0.61±0.001	15.38±0.82**	3.05±0.71*	2.75±0.21	3.99±0.17**	0.19±0.01	6.90±0.18**	0.69±0.23	17.57±0.82**

The values with± shows the standard error of mean values of three replications. The asterisks (

*p<0.05

**p<0.01

***p<0.001) show that the values are significantly different in each column using a One-Way ANOVA analysis in Graph Pad prism (v6.01) using Bonferroni test

The HPLC results reported herein are in total agreement with our recently published quantification of KBA (keto-β-boswellic acid) and AKBA (3-acetyl-11-keto-β-boswellic acid) using NIRS coupled with PLS regression [73,40]. For the first time, NIR spectroscopy coupled with PLS regression as a rapid and alternative method was developed to quantify the amount of AKBA and KBA in different plant parts of *Boswellia sacra* and the resin exudates of the trunk. The quantification of various plant parts and the resin indicated that the MeOH extract of the resin has the highest concentration of AKBA (7.0%) followed by epidermis (1.37%), and essential oil (0.1%). Thus it can be concluded that AKBA is only present in the gum-resin exudate and epidermis of the stem. The existence of moderate amounts of AKBA in the epidermis is due to the presence of resin-producing canals [40]. A similar study was conducted for KBA and obtain results indicated that the MeOH extract of resin has the highest concentration of KBA (0.6%) followed by essential oil (0.1%). The MeOH extract of the resin was subjected to column chromatography to get various sub-fractions at different polarity of organic solvents. The sub-fraction at 4% MeOH/CHCl_3_ (4.1% of KBA) was found to contain the highest percentage of KBA followed by another sub-fraction at 2% MeOH/CHCl_3_ (2.2% of KBA; Table 2). The results also indicated that KBA is only present in the gum-resin of the trunk and not in the plant itself [73].

**Fig 8:**
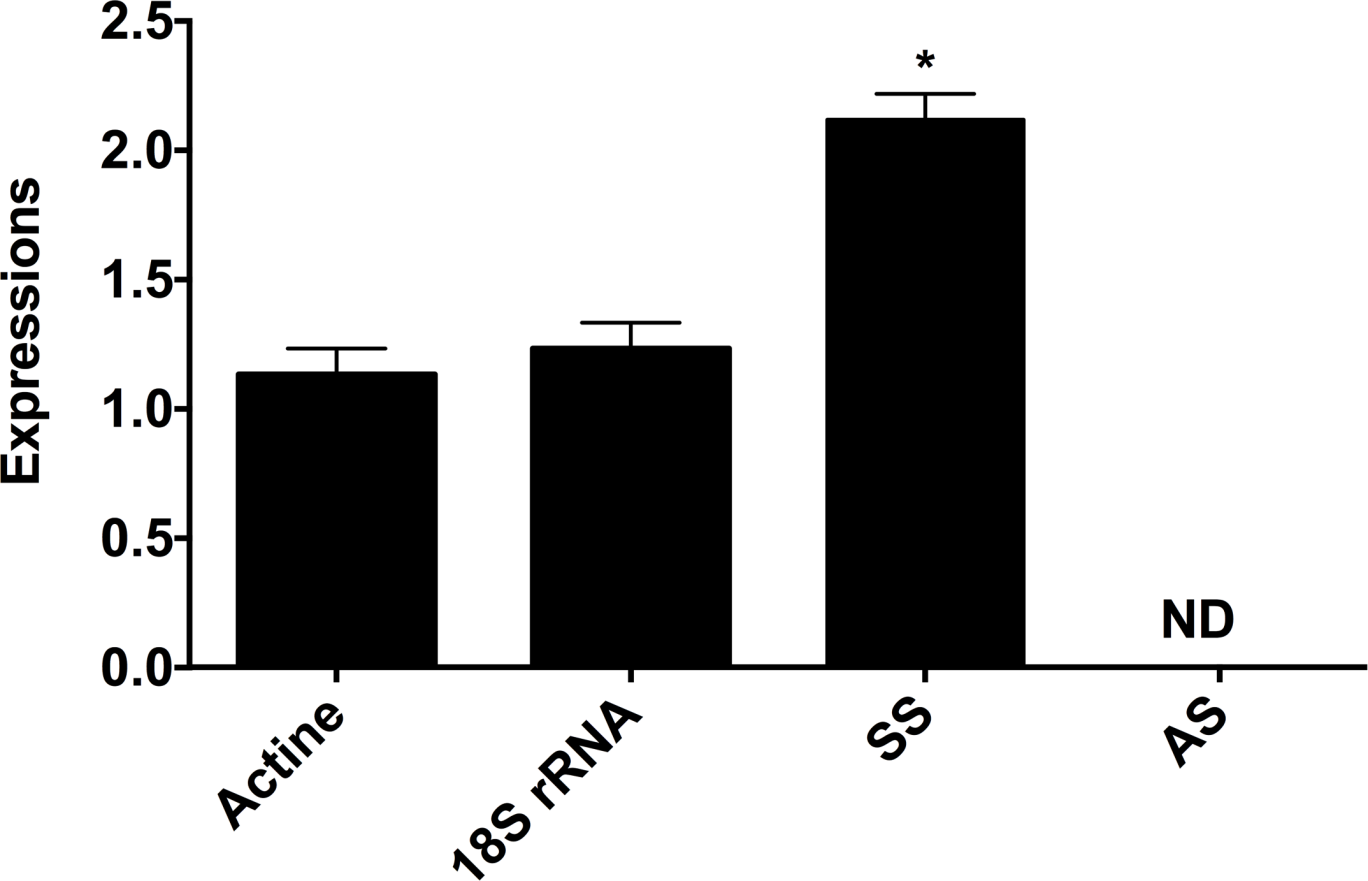
RT-PCR analysis of α*-amyrin synthase* (AS) and *Squalene-synthase* (SS) in the leaf part of *B*. *sacra*. The values are mean of three replications and bars with * shows values are significantly (P<0.05) different from the expression of Actin.

Interestingly, there are clear differences with regard to the quantities of amyrins and boswellic acids of the two grades of *B*. *sacra* which explain the influence of the geographical location and the environmental conditions of the two grades. This is in agreement with our recent genetic diversity work, which was based on the analysis of simple sequences repeat, and random-amplified polymorphic DNA genetic markers, suggesting a clear isolation of *B*. *sacra* populations in the eastern coastal regions (unpublished data). Thus, the current variation in the results can also be attributed to the tree’s genetic diversity and climatic conditions. This is also in correlation with the results published [74–76] where it was shown that both genetic and climatic factors can influence the resin production and composition. To further confirm expression of amyrin related transcripts, primers were designed using known available genomic sets derived from *Azadirachta indica*. Since biochemical related genomic and molecular information are not available for any of *Boswellia* species, therefore, the homologues species at order level (*Azadirachta indica*) was selected [77]. The gene expression for both α*-amyrin synthase* was not detected in the leaf part of the *B*. *sacra* tree (Fig 8). However, *Squalene-synthase* related transcript was expressed in leaf part which suggests that BAs might be synthesized through this precursor. However, this needs a detailed transcriptomic analysis to obtain conclusive evidences on the biosynthetic pathway.

**Fig 9:**
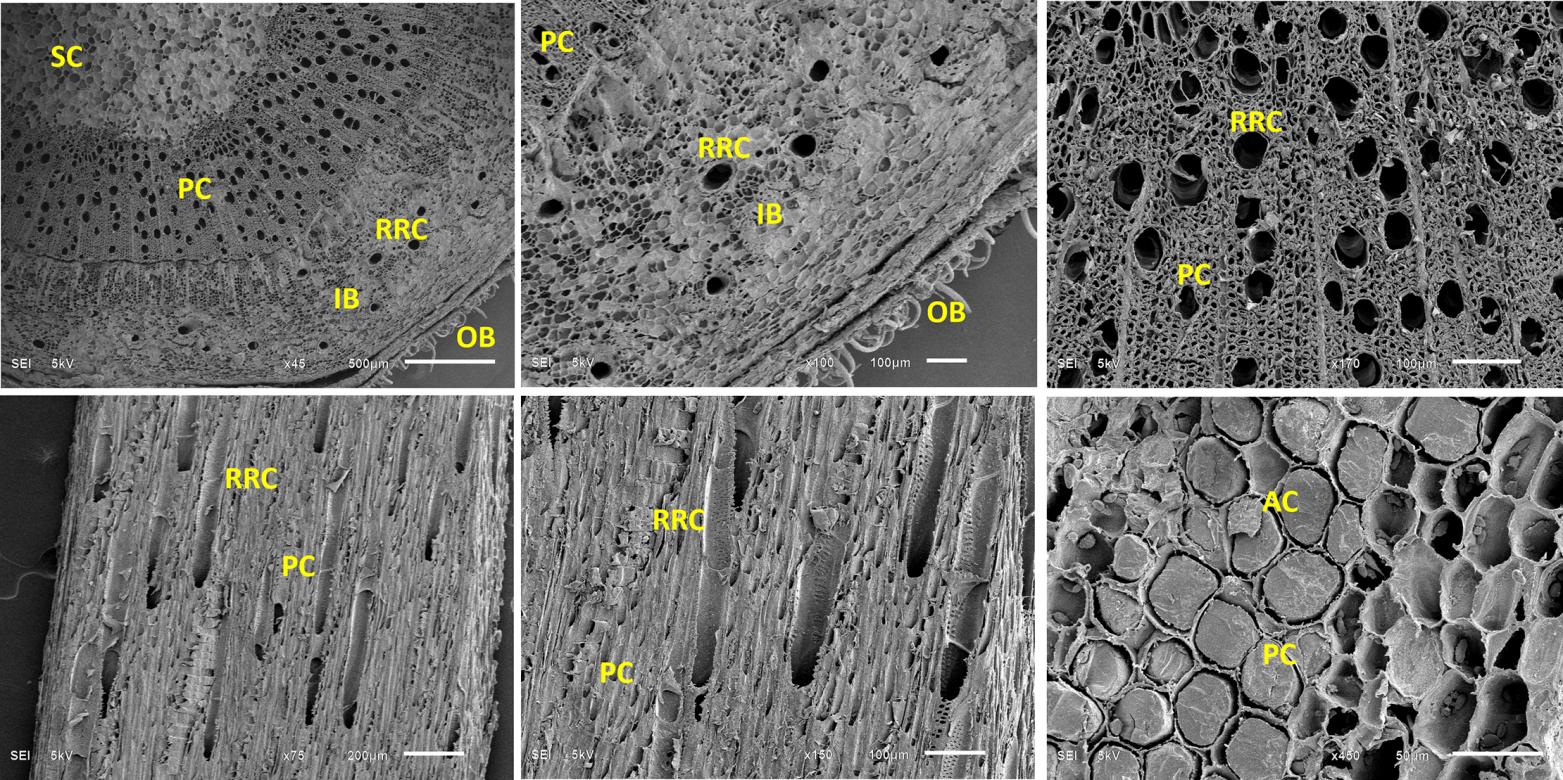
Transversal and radial sectioning of *B*. *sacra* stem part structure and analysis through Scanning Electron Micrographs. The micrographs are representative to ten individual stem parts of the tree. The Micrographs are taken on SEM using 50 to 200 μM section and zoom from 100x to 1.2Kx. SC = Sclerenchyma; PC = Parenchyma; AC = Axial canals; RRC = Radial resin canal; IB = Inner bark; OB = Outer bark.

Interestingly, the roots contain α-amyrin (2.63%) and proved a complete absence of β-amyrin and all boswellic acids. The leaves have only trace amounts of β-ABA and β-AKBA. Not unexpectedly, the stem proved to be completely free of amyrins and boswellic acids. This explains that the production of the resin is taking place at the cambium (total boswellic acids 1.0%) and epidermis (total of boswellic acids 4.93%) due to the existence of the resin-producing canals. This is supported by the scanning electron microscope images (Fig 9). The micrographs clearly demarcate the presence of radial and axil resin canals in the inner bark region (between epidermis and vascular cambium). The current results show an anatomical harmony with the presence of resinous ducts in the *B*. *papyrifera* [78].

## Conclusion

The current study showed the isolation and characterization of new chemical constituents (β-boswellic aldehyde and 3β, 11β-dihydroxy BA) from the *B*. *sacra* resins along with known α-amyrin (3-*epi*-α-amyrin, β-amyrin and α-amyrin). A detailed elucidation of the BAs biosynthesis was made for the first time, providing chemical, molecular and structural details to reinforce the previously reported studies. A detailed quantification of BAs and their amyrin precursors in different parts of three *Boswellia* species (*B*. *sacra*, *B*. *serrata* and *B*. *papyrifera*) suggests variation of triterpenes on the basis of species, tree parts and geographical locations. However, BAs were more consistently available in the inner bark of the tree. This was also revealed by the micrographs of resin canals in the inner bark. The current study concludes that BAs are the major class of chemical constituents in *Boswellia*, where future studies should consider the characterization of enzymes responsible in its biosynthesis.

## References

[1] Raffaelli M, Mosti M, Tardelli M. *Boswellia sacra* Flueck. (Burseraceae) in the Hasik area (eastern Dhofar, Oman) and a list of the surrounding flora. Webbia 2006; 61:245–251.

[2] Raffaelli M, Mosti S, Tardelli M. The Frankincense Tree (*Boswellia sacra* Flueck., Burseraceae) in Dhofar, southern Oman: field-investigations on the natural populations. Webbia 2003; 58:133–149.

[3] Coppi A, Cecchi L, Selvi F, Raffaelli M. The frankincense tree (*Boswellia sacra*, Burseraceae) from Oman: ITS and ISSR analyses of genetic diversity and implications for conservation. Genet Resour Crop Evol. 2010; 57:1041–1052.

[4] Al-Harrasi A, Ali L, Hussain J, Rehman NU, Mahjabeen, Ahmed M, et al Analgesic effects of crude extracts and fractions of Omani frankincense obtained from traditional medicinal plant *Boswellia sacra* on animal models. Asian Pac J Trop Med. 2014; 7(Suppl 1):S485–S490. 10.1016/S1995-7645(14)60279-0 25312172

[5] Asad M, Alhumoud M. Hepatoprotective effect and GC-MS analysis of traditionally used *Boswellia sacra* oleo gum resin (Frankincense) extract. Afr J Tradit Complement. 2015; 12:1–5.

[6] Al-Harrasi A, Ali L, Ceniviva E, Al-Rawahi A, Hussain J, Hussain H, et al Antiglycation and antioxidant activities and HPTLC analysis of *Boswellia sacra* oleogum resin: The sacred frankincense. Trop J Pharm Res. 2013; 12:597–502.

[7] Yassin NAZ, El-Shenawy SMA, Mahdy KA, Gouda NAM, Marrie AEH, Farrag ARH, et al Effect of *Boswellia serrata* on Alzheimer’s disease induced in rats. J Arab Soc Med Res. 2013; 8:1–11.

[8] Asif M, Jabeen Q, Majid AMSA, Atif M. Diuretic activity of *Boswellia serrata* Roxb. oleo gum extract in albino rats. Pak J Pharm. Sci. 2014; 27:1811–1817. 25362605

[9] Al-Harrasi A, Ali L, Rehman NU, Hussain J, Hussain H, Al-Rawahi A, et al 11α-Ethoxy-β-boswellic acid and nizwanone, a new boswellic acid derivative and a new triterpene respectively, from *Boswellia sacra*, Chem Biodivers. 2013; 10:1501–1506. doi: 10.1002/cbdv.201200231 23939798 10.1002/cbdv.201200231

[10] Ni X, Suhail MM, Yang Q, Cao A, Fung KM, Postier RG, et al Frankincense essential oil prepared from hydrodistillation of *Boswellia sacra* gum resins induces human pancreatic cancer cell death in cultures and in a xenograft murine model, BMC Complement Altern Med. 2012; 12:253 doi: 10.1186/1472-6882-12-253 23237355 10.1186/1472-6882-12-253 PMC3538159

[11] Pan YN, Liang XX, Niu LY, Wang YN, Tong X, Hua HM, et al Comparative studies of pharmacokinetics and anticoagulatory effect in rats after oral administration of Frankincense and its processed products. J Ethnopharmacol. 2015; 172:118–123. doi: 10.1016/j.jep.2015.06.029 26117531 10.1016/j.jep.2015.06.029

[12] Suhail MM, Wu W, Cao A, Mondalek FG, Fung KM, Shih PT, et al *Boswellia sacra* essential oil induces tumor cell-specific apoptosis and suppresses tumor aggressiveness in cultured human breast cancer cells. BMC Complement Altern Med. 2011; 11:1–14. doi: 10.1186/1472-6882-11-1 22171782 10.1186/1472-6882-11-129 PMC3258268

[13] Banno N, Akihisa T, Yasukawa K, Tokuda H, Tabata K, Nakamura Y, et al Anti-inflammatory activities of the triterpene acids from the resin of *Boswellia carteri*. J Ethnopharmacol. 206; 107:249–253. 10.1016/j.jep.2006.03.006 16621377

[14] Zaki AA, Hashish NE, Amer MA, Lahloub MF. Cardioprotective and antioxidant effects of oleogum resin “Olibanum” from *Boswellia carteri* Birdw. (Bursearceae). Chin J Nat Med. 2014; 12:345–350. doi: 10.1016/S1875-5364(14)60042-X 24856757 10.1016/S1875-5364(14)60042-X

[15] Asad M, Alhomoud M. Proulcerogenic effect of water extract of *Boswellia sacra* oleo gum resin in rats. Pharm Biol. 2016; 54:225–230. doi: 10.3109/13880209.2015.1028553 25853959 10.3109/13880209.2015.1028553

[16] Tolera M, Sass-Klaassen U, Eshete A, Bongers F, Sterck FJ. Frankincense tree recruitment failed over the past half century. For Ecol Manage. 2013; 304:65–72.

[17] Tadesse W, Desalegn G, Alia R. Natural gum and resin bearing species of Ethiopia and their potential applications. Investigación agrarian: Sistemas y Recursos Forestales 2007; 16:211–221.

[18] Addisalem AB, Bongers F, Kassahun T, Smulders MJM. Genetic diversity and differentiation of the frankincense tree (*Boswellia papyrifera* (Del.) Hochst) across Ethiopia and implications for its conservation. For Ecol Manag. 2016; 360:253–260.

[19] Eshete A, Sterck FJ, Bongers F. Frankincense production is determined by tree size and tapping frequency and intensity. For Ecol Manag. 2012; 274:136–142.

[20] Mengistu T, Sterck FJ, Anten NPA, Bongers F. Frankincense tapping reduced photosynthetic carbon gain in *Boswellia papyrifera* (Burseraceae) trees. For Ecol Manag. 2012; 278:1–8.

[21] Farah MH. Non-timber forest product (NTFP) extraction in arid environments: land-use change, frankincense production and the sustainability of *Boswellia sacra* in Dhofar (Oman). The University of Arizona; 2008.

[22] Rees AR. Frankincense and myrrh. New Plantsman 1995; 2:55–59.

[23] Coppi A, Cecchi L, Selvi F, Raffaelli M. The Frankincense tree (*Boswellia sacra*, Burseraceae) from Oman: ITS and ISSR analyses of genetic diversity and implications for conservation. Genet Resour Crop Evol. 2010; 57:1041–1052.

[24] Hammer K, Gebauer J, Al Khanjari S, Buerkert A. Oman at the cross-roads of inter-regional exchange of cultivated plants. Genet Resour Crop Evol. 2009; 56:547–560.

[25] Kreck C, Saller R. Indischer Weihrauch und seine Zubereitungen einschliesslich H15 als traditionelle und moderne Therapeutika. Internist Prax. 1998; 857–872.

[26] Shen T, Lou HX. Bioactive constituents of myrrh and frankincense, two simultaneously prescribed gum resins in Chinese traditional medicine. Chem Biodivers. 2008; 5:540–553. doi: 10.1002/cbdv.200890051 18421746 10.1002/cbdv.200890051

[27] Shah BA, Qazi GN, Taneja SC. Boswellic acids: a group of medicinally important compounds. Nat Prod Rep. 2009; 26:72–89. 19374123 10.1039/b809437n

[28] Roy NK, Deka A, Bordoloi D, Mishra S, Kumar AP, Sethi G, et al The potential role of boswellic acids in cancer prevention and treatment. Cancer Lett. 2016; 377:74–86. doi: 10.1016/j.canlet.2016.04.017 27091399 10.1016/j.canlet.2016.04.017

[29] Hussain H, Al-Harrasi A, Csuk R, Shamraiz U, Green IR, Ahmed I. Therapeutic potential of boswellic acids: a patent review (1990–2015). Expert Opin Ther Pat. 2017; 27:81–90. doi: 10.1080/13543776.2017.1235156 27646163 10.1080/13543776.2017.1235156

[30] Winterstein A, Stein G. Untersuchungen in der Saponinreihe X. zur kenntnis der mono-oxy-triterpenesäuren. Z physiol Chem. 1932; 208:9–25.

[31] Ruzicka L, Wirz W. Zur kenntnis der triterpene (50. Mitteilung). umwandlung der ß-boswellinsäure in alpha-amyrin. Helv Chim Acta. 1939; 22:948–951 (1939).

[32] Ruzicka L, Wirz W. Zur kenntnis der triterpene (52. Mitteilung). umwandlung der alpha-boswellinsäure in β-amyrin. Helv Chim Acta.1940; 23:132–135.

[33] Beton JL, Halsall TG, Jones ERH. The chemistry of triterpenes and related compounds. Part XXVIII. β-boswellic acid. J Chem Soc. 1956; 2904–2909.

[34] Pardhy RS, Bhattacharyya SC. β-Boswellic acid, Acetyl-ß-boswellic acid, Acetyl-11-keto-ß-boswellic acid and 11-Keto-ß-boswellic acid, four pentacyclic triterpene acids from the resin *Boswellia serrata* Roxb. Indian J Chem. 1978; 16B:176–178.

[35] Belsner K, Büchele B, Werz U, Syrovets T, Simmet T. Structural analysis of pentacyclic triterpenes from the gum resin of *Boswellia serrata* by NMR spectroscopy. Magn Reson Chem. 2003; 41:115–122.

[36] Schweizer S, Eichele K, Ammon HP, Safayhi H. 3-Acetoxy group of genuine AKBA (acetyl-11-keto-beta-boswellic acid) is alpha-configurated. Planta Med. 2000; 66:781–782. doi: 10.1055/s-2000-9614 11199146 10.1055/s-2000-9614

[37] Schweizer S, von Brocke AF, Boden SE, Bayer E, Ammon HP, Safayhi H. Workup-dependent formation of 5-lipoxygenase inhibitory boswellic acid analogues. J Nat Prod. 2000; 63:1058–1061. 10978197 10.1021/np000069k

[38] Sailer ER, Schweizer S, Boden SE, Ammon H, Safayhi H. Characterization of an acetyl‐11‐keto‐β‐boswellic acid and arachidonate‐binding regulatory site of 5‐lipoxygenase using photoaffinity labeling. The FEBS Journal 1998; 256:364–368. 10.1046/j.1432-1327.1998.2560364.x 9760176

[39] Ammon HP. Boswellic acids (components of frankincense) as the active principle in treatment of chronic inflammatory diseases. Wien Med Wochenschr (1946). 2002; 152: 373–378. 12244881 10.1046/j.1563-258x.2002.02056.x

[40] Johann J, Bergmann J. An efficient method for the large‐scale preparation of 3‐O‐acetyl‐11‐oxo‐β‐boswellic acid and other Boswellic acids. Eur J Org Chem. 2003; 2003:4752–4756.

[41] Henkel A, Kather N, Mönch B, Northoff H, Jauch J, Werz O. Boswellic acids from frankincense inhibit lipopolysaccharide functionality through direct molecular interference. Biochem Pharmacol. 2012; 83:115–121. doi: 10.1016/j.bcp.2011.09.026 22001311 10.1016/j.bcp.2011.09.026

[42] Verhoff M, Seitz S, Paul M, Noha SM, Jauch J, Schuster D, et al Tetra-and pentacyclic triterpene acids from the ancient anti-inflammatory remedy frankincense as inhibitors of microsomal prostaglandin E2 synthase-1. J Nat Prod. 2014; 77:1445–1451. doi: 10.1021/np500198g 24844534 10.1021/np500198g PMC4074212

[43] Al-Harrasi A, Rehman NU, Mabood F, Albroumi M, Ali L, Hussain J, et al Application of NIRS coupled with PLS regression as a rapid, non-destructive alternative method for quantification of KBA in *Boswellia sacra*. Spectrochim Acta Mol Biomol Spectrosc. 2017; 184:277–285. 10.1016/j.saa.2017.05.018 28525862

[44] Rehman NU, Ali L, Al-Harrasi A, Mabood F, Al-Broumi M, Khan AL, et al Quantification of AKBA in *Boswellia sacra* using NIRS coupled with PLS regression as alternative method and cross validation by HPLC, Phytochem Anal. 2018; 29:137–143. doi: 10.1002/pca.2721 28881407 10.1002/pca.2721

[45] Wolfram RK, Barthel-Niesen A, Schäfer R, Heller L, Al-Harrasi A, Csuk R. Synthesis and cytotoxic screening of beta-boswellic acid derivatives. Mediterr J Chem. 2017; 6:142–164.

[46] Mannino G, Occhipinti A, Maffei ME. Quantitative determination of 3-O-Acetyl-11-Keto-β-Boswellic Acid (AKBA) and other boswellic acids in *Boswellia sacra* Flueck (syn. *B*. *carteri Birdw*) and *Boswellia serrata* Roxb. Molecules 2016; 21:1329. 10.3390/molecules21101329 PMC6273064 27782055

[47] Reising K, Meins J, Bastian B, Eckert G, Mueller WE, Schubert-Zsilavecz M, et al Determination of boswellic acids in brain and plasma by high-performance liquid chromatography/tandem mass spectrometry. Anal Chem. 2005; 77:6640–6645. doi: 10.1021/ac0506478 16223251 10.1021/ac0506478

[48] Ikuro A. Enzymatic synthesis of cyclic triterpenes, Nat Prod Rep. 2007; 24:1311–1331. doi: 10.1039/b616857b 18033581 10.1039/b616857b

[49] Chen GJ, Jin S, Goodwin PH. An improved method for the isolation of total RNA from *Malva pusilla* tissues infected with *Colletotrichum gloeosporioides*. J Phytopathol. 2000; 148:57–60.

[50] Peckys DB, Baudoin JP, Eder M, Werner U, De Jonge N. Epidermal growth factor receptor subunit locations determined in hydrated cells with environmental scanning electron microscopy. Sci Rep. 2013; 3:2626 doi: 10.1038/srep02626 24022088 10.1038/srep02626 PMC3769654

[51] Manguro LOA, Wagai SO, Onyango JO. Terpenoids of *Boswellia neglecta* oleo-gum resin. Bull Chem Soc Ethiop. 2016; 30:317–323.

[52] Taneja SC, Mahajan B, Sethi VK, Dhar KL. Two triterpenoids from *Boswellia serrata* gum resin. Phytochemistry 1995; 39:453–455.

[53] Badria FA, Mikhaeil BR, Maatooq GT, Amer MMA. Immunomodulatory triterpenoids from the oleogum resin of *Boswellia carterii* Birdwood. Z Naturforsch. 2003; 58c:505–516. 10.1515/znc-2003-7-811 12939036

[54] Lima FV, Malheiros A, Otuki MF, Calixto JB, Yunes RA, Filho VC, et al Three new triterpenes from the resinous bark of *Protium kleinii* and their antinociceptive activity. J. Braz Chem Soc. 2005; 16:578–582.

[55] Basar, S. Phytochemical Investigations on Boswellia Species, PhD Thesis, University of Hamburg, Hamburg, Germany; 2005.

[56] Zhao M, Zhang S, Fu L, Li N, Bai J, Sakai J, et al Taraxasterane- and ursane-type triterpenes from *Nerium oleander* and their biological activities. J Nat Prod. 2006; 69:1164–1167. doi: 10.1021/np0680073 16933868 10.1021/np0680073

[57] Cseke LJ, Kirakosyan A, Kaufman PB, Warber S, James A. Duke JA, et al Natural Products from Plants Taylor & Francis: Boca Raton, FL, USA; 2006.

[58] Manguro LOA, Wagai SO. Ursane and tirucallane-type triterpenes of *Boswelli arivae* oleo-gum resin. J Asian Nat Prod Res. 2016; 18:854–864. doi: 10.1080/10286020.2016.1165674 27049028 10.1080/10286020.2016.1165674

[59] Shah BA, Kumar A, Gupta P, Sharma M, Sethi VK, Saxena AK, et al Cytotoxic and apoptotic activities of novel amino analogues of boswellic acids. Bioorg Med Chem Lett. 2007; 17:6411–6416. doi: 10.1016/j.bmcl.2007.10.011 17950603 10.1016/j.bmcl.2007.10.011

[60] Singh SK, Tripathi VJ. A new pentacyclic triterpene acid from *Lantana indica*. J Nat Prod. 1999; 54:755–758.

[61] Greve HL, Kaiser M, Brun R, Schmidt TJ. Terpenoids from the oleo-gum-resin of *Boswellia serrate* and their antiplasmodial effects in vitro. Planta Med. 2017; 83:1214–1226. doi: 10.1055/s-0043-116943 28738439 10.1055/s-0043-116943

[62] Fatcorusso E, Santacroce C, Xaasan CF. Damamrnae triterpenes from the resin of *Boswellia freerana*. Phytochemistry 1985; 24:1035–1036.

[63] Eschenmoser A, Ruzicka L, Jeger O, Arigoni D. Zur kenntnis der triterpene. 190. mitteilung. eine stereochemische interpretation der biogenetischen isoprenregel bei den Triterpenen. Helv Chim Acta. 1955; 38:1890–1904.

[64] Eschenmoser A, Arigoni D. Revisited after 50 Years: The stereochemical interpretation of the biogenetic isoprene rule for the triterpenes. Helv Chim Acta. 2005; 88:3011–3050.

[65] Pardhy RS, Bhattacharyya SC. Tetracyclic triterpene acids from the resin of *Boswellia serrata* Roxb. Ind J Chem. 1978; 16B:174–175.

[66] Proietti G, Strapaghetti G, Corsano S. Triterpenes of *Boswellia frereana*. Planta Med. 1981; 41:417–418. doi: 10.1055/s-2007-971743 17401871 10.1055/s-2007-971743

[67] Mannino G, Occhipinti A, Maffei ME. Quantitative determination of 3-O-Acetyl-11-Keto-β-Boswellic Acid (AKBA) and other Boswellic acids in *Boswellia sacra* Flueck (syn. B. carteri Birdw) and *Boswellia serrata* Roxb. Molecules 2016; 21:1329. 10.3390/molecules21101329 PMC6273064 27782055

[68] Reddy VLN, Ravinder K, Srinivasulu M, Goud TV, Reddy SM, Srujankumar D, et al Two new macrocyclic diaryl ether heptanoids from *Boswellia ovalifoliolata*. Chem Pharm Bull. 2003; 51:1081–1084. 12951452 10.1248/cpb.51.1081

[69] Dekebo A, Dagne E, Gautum OR, Aasen AJ. Triterpenes from the resin of Boswellia neglecta. B Chem Soc Ethiopia 2002; 16:87–90.

[70] Pardhy RS, Bhattacharyya SC. β-Boswellic acid, Acetyl- β-boswellic acid, Acetyl-11-keto- β-boswellic acid and 11-Keto- β-boswellic acid, Four pentacyclic triterpene acids from the resin of *Boswellia serrata* Roxb. Ind J Chem. 1978; 16B:176–178.

[71] Culioli G, Mathe C, Archier P, Vieillescazes C. A lupane triterpene from frankincense (*Boswellia* sp., Burseraceae). Phytochemistry 2003; 62:537–541. 12560022 10.1016/s0031-9422(02)00538-1

[72] Al-Harrasi A, Ali L, Rehman NU, Hussain H, Hussain J, Al-Rawahi A, et al Nine triterpenes from *Boswellia sacra* and their chemotaxonomic importance. Biochem Syst Ecol. 2013; 51:113–116.

[73] Poeckel D, Tausch L, Kather N, Jauch J, Werz O. Boswellic acids stimulate arachidonic acid release and 12-lipoxygenase activity in human platelets independent of Ca^2+^ and differentially interact with platelet-type 12-lipoxygenase. Mol Pharmacol. 2006; 70:1071–1078. doi: 10.1124/mol.106.024836 16788089 10.1124/mol.106.024836

[74] Addisalem AB, Bongers F, Kassahun T, Smulders MJM. Genetic diversity and differentiation of the frankincense tree (*Boswellia papyrifera* (Del.) Hochst) across Ethiopia and implications for its conservation. For Ecol Manag. 2016; 360:253–260.

[75] Lemenih M, Abebe T, Olsson M. Gum and resin resources from some Acacia, *Boswellia* and Commiphora species and their economic contributions in Liban, south-east Ethiopia. J Arid Environ. 2003; 55:465–482.

[76] Olate VR, Soto A, Schmeda-Hirschmann G. Seasonal variation and resin composition in the Andean tree *Austrocedrus chilensis*. Molecules 2014; 19:6489–6503. doi: 10.3390/molecules19056489 24853713 10.3390/molecules19056489 PMC6271173

[77] Khan AL, Al-Harrasi A, Asaf S, Park CE, Park GS, Khan AR et al The First Chloroplast Genome Sequence of *Boswellia sacra*, a Resin-Producing Plant in Oman. PLoS One 2017; 12:e0169794 doi: 10.1371/journal.pone.0169794 28085925 10.1371/journal.pone.0169794 PMC5235384

[78] Tolera M, Menger D, Sass-Klaassen U, Sterck FJ, Copini P, Bongers F. Resin secretory structures of *Boswellia papyrifera* and implications for frankincense yield. Ann Bot. 2013; 111:61–68. doi: 10.1093/aob/mcs236 23223203 10.1093/aob/mcs236 PMC3523649

